# 

**Published:** 1871-02

**Authors:** 


### American Medical Association.

Office of Permanent Secretary, Wm. B. Atkinson, M. D.,
1400 Pine Street, S. W. cor. Brord, Philadelphia.

The Twenty-second Annual Session will be held in San Francisco, Cal., May 2, 1871, at 11 A. M.

Secretaries of all medical organizations are requested to forward lists of their Delegates as soon as elected, to the Permanent Secretary.

☞ Any respectable physician who may desire to attend, but cannot do so as a delegate, may be made a member by invitation, upon the recommendation of the Committee of Arrangements.

W. B. ATKINSON.

### Books and Pamphlets Received.

Transactions of the Medical Society of the State of New York.

The Journal of the Gynaecological Society of Boston, Vol. III.

Transactions of the New York State Eclectic Medical Society, Vol. IV.

Transactions of the American Ophthalmological Society. Seventh Annual Meeting.

The Opthalmoscope in the treatment of Epilepsy. By Reuben A. Vance, M. D.

Report of the Board of Health of the City of Chicago, and a Sanitary history of Chicago from 1833 to 1870.

American Association for the Cure of Inebriates Proceedings of the First Meeting.

Annual Report of the New York State Inebriate Asylum at Binghamton, N. Y., for the year 1870.

Memorial of the Prison Association to the Governor of the State of New York.

The Atlantic Monthly; The Nation; Peters' Musical Monthly; New York Observer; Scientific American.

